# The Role of Additional Pulses in Electropermeabilization Protocols

**DOI:** 10.1371/journal.pone.0113413

**Published:** 2014-12-01

**Authors:** Cecilia Suárez, Alejandro Soba, Felipe Maglietti, Nahuel Olaiz, Guillermo Marshall

**Affiliations:** 1 Laboratorio de Sistemas Complejos, Departamento de Computación, Facultad de Ciencias Exactas y Naturales, Universidad de Buenos Aires, Buenos Aires, Argentina; 2 Centro de Simulación Computacional - CONICET y Comisión Nacional de Energia Atómica, Buenos Aires, Argentina; University of California at Berkeley, United States of America

## Abstract

Electropermeabilization (EP) based protocols such as those applied in medicine, food processing or environmental management, are well established and widely used. The applied voltage, as well as tissue electric conductivity, are of utmost importance for assessing final electropermeabilized area and thus EP effectiveness. Experimental results from literature report that, under certain EP protocols, consecutive pulses increase tissue electric conductivity and even the permeabilization amount. Here we introduce a theoretical model that takes into account this effect in the application of an EP-based protocol, and its validation with experimental measurements. The theoretical model describes the electric field distribution by a nonlinear Laplace equation with a variable conductivity coefficient depending on the electric field, the temperature and the quantity of pulses, and the Penne's Bioheat equation for temperature variations. In the experiments, a vegetable tissue model (potato slice) is used for measuring electric currents and tissue electropermeabilized area in different EP protocols. Experimental measurements show that, during sequential pulses and keeping constant the applied voltage, the electric current density and the blackened (electropermeabilized) area increase. This behavior can only be attributed to a rise in the electric conductivity due to a higher number of pulses. Accordingly, we present a theoretical modeling of an EP protocol that predicts correctly the increment in the electric current density observed experimentally during the addition of pulses. The model also demonstrates that the electric current increase is due to a rise in the electric conductivity, in turn induced by temperature and pulse number, with no significant changes in the electric field distribution. The EP model introduced, based on a novel formulation of the electric conductivity, leads to a more realistic description of the EP phenomenon, hopefully providing more accurate predictions of treatment outcomes.

## Introduction

Exposure of biological membranes to specific pulsed electric fields induces the rise (permanent or transient) of membrane permeability, which is called electropermeabilization (EP) or electroporation. EP phenomenon is exploited in electrochemotherapy (ECT) [1]–[3]), gene electrotransfer (GET) [4]–[6]), irreversible electroporation (IRE) [7] and nanoelectroporation (NAP) [8], among others. EP-based technologies are at present being applied to a broad spectrum of biotechnological fields, including not only medical applications but also food processing and environmental management [9]–[11]. All of them share the application of electric pulses to permeabilize cell membranes, although the actual formation of pores during the process (electroporation) is at present a matter of research [12].

ECT is one of the most explored approach among all EP techniques, consisting in the transient permeabilization of the cell membrane to let the introduction of a specific drug (usually bleomycin or cisplatin) with much more efficiency into the target cell. This treatment is nowadays of standard clinical use in Europe for cutaneous and subcutaneous tumors [13],[14]. The amount of permeabilization, and thus the efficiency of the EP protocol, depends on the local electric field but also on tissue electric conductivity [15] [16]. It was also experimentally established that, for certain EP protocols, multiple pulses rise this conductivity [15]. Even more, it has been observed that the number of pulses applied also affect the amount of permeabilization [17]–[21].

Besides animal tissues, simple fruit and vegetable tissue models (specially potato tuber) have been used to study the effects of EP treatments as electric field redistribution [22], metabolic changes [23] and tissue damage [24]–[25]. In potato tuber, these damages occur through oxidation processes due to the release of intracellular enzymes (mainly polyphenol oxidases) that are evidenced by brown-black areas [24]. As the release of these enzymes implies the permeabilization of the cell membrane, dark areas resulting from an EP treatment in this tissue may be considered as electropermeabilized.

Treatment planning of EP-based medical interventions in tumors by numerical modeling has been evolving during the last decade tending to ensure that only targeted regions were treated, sparing as far as possible normal tissue that is meant to be preserved [26]–[28]. Initial models consider conductivity as a constant parameter derived from tissue characteristics, but soon it was evident that conductivity significantly changes during the electroporation process and models evolve to include a non-linear conductivity first dependent on the electric field [29]–[32], and later on the electric field and the temperature [33],[34].

In general, it is recognized that conductivity changes during ECT are due to an increment in membrane conductance, a phenomenon that may occur mainly during the length-time of the first pulse [22]. This membrane permeabilization would increase two transport processes: electrophoresis during the pulse and diffusion as long as the membrane remains permeabilized [16],[17]. Multiple pulses may also increase membrane conductance [20] as well as sequentially recruit more electropermeabilized cells of the treated area thus increasing tissue electric conductivity.

The goal of this paper is to introduce a new conductivity formulation dependent on the electric field, the temperature and the train of pulses, and to show its effects in electroporation protocols. We called this formulation pulse-dependent-conductivity. We also present experimental results derived from potato tuber that validate this new formulation.

## Methods

### 0.1 *In vitro* model

**Figure 1:**
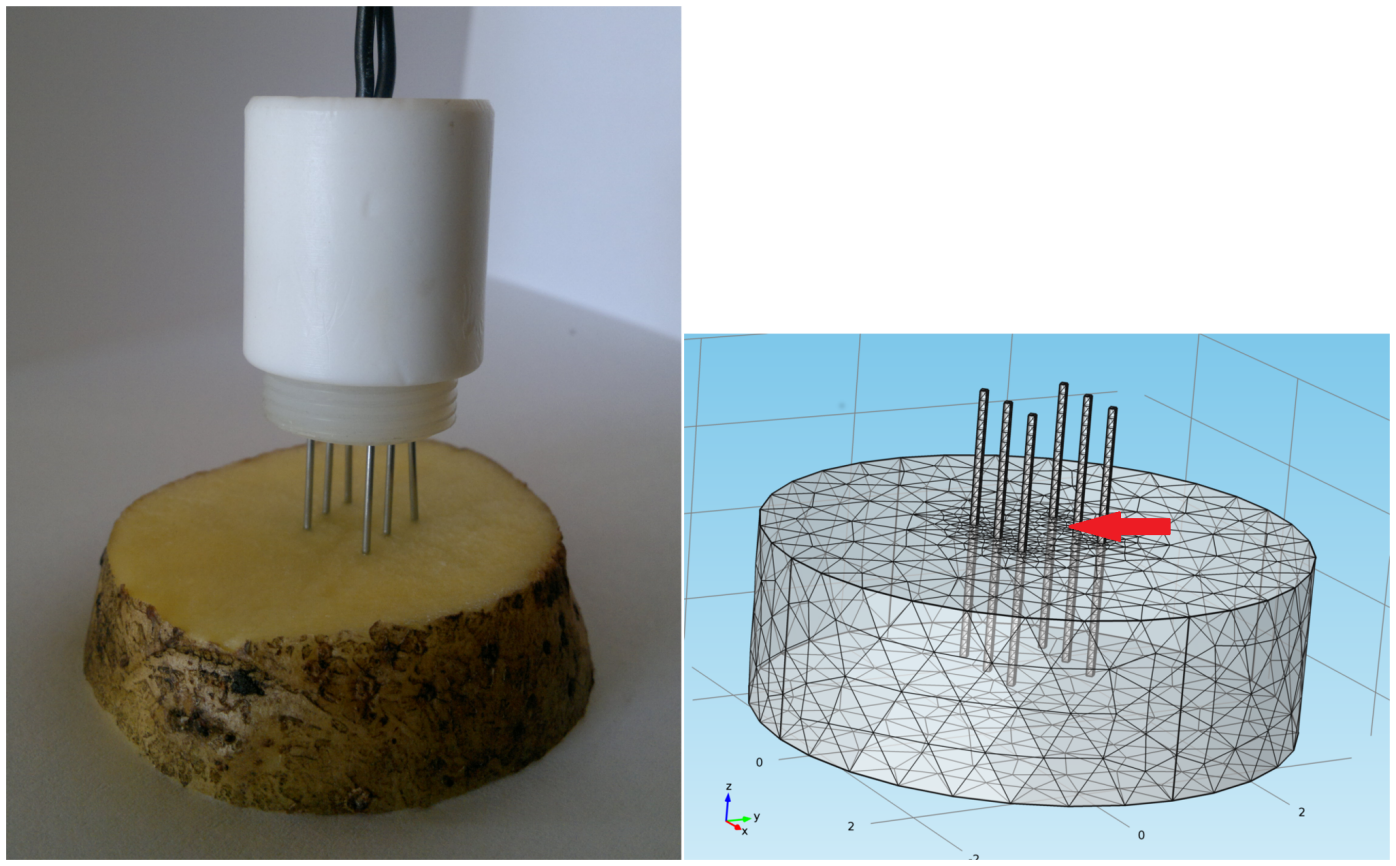
In vitro and in silico models. a) Experimental setup. Arrangement of six electrodes inserted in a potato slice. b) Domain and mesh of the mathematical model generated by Comsol. The arrow indicates the point where all calculations presented in figure 2 were made (measurement area).

Experimental setup is based in the application of different EP protocols to a vegetal tissue consisting in potato tuber (*Solanum tuberosum sp.*) and the measurement of electric currents as well as electropermeabilized areas during the treatments. Potato tuber slices (4×5×2 *cm* ^3^) were electroporated with an arrange of three pair of parallel surgical steel electrodes 2 cm long and 1 mm diameter, immersed 1 cm in the tissue, with 1 cm from anode to cathode and 0.5 cm between two electrodes of the same polarity (a typical type II configuration used in clinics [13],[35]). Figure 1a shows the used experimental setup.

Twenty four square electric pulses (voltages of 500, 800, 1000, 1500 and 1700 V with a duration of 100 *µ*s at 1 Hz) were applied by means of a square wave electroporator (BTX ECM 830, Harvard Apparatus Inc, MA, USA). Pulse amplitudes were chosen to correspond to those used in standard ECT and IRE protocols. Total electric currents were recorded all along the treatment by an oscilloscope DSOX2012A, 100 MHz, 2 channels (Agilent Technologies, CA, USA) connected to the wire powering the 3 cathodes. Twelve hours after treatment, potato slices were photographed and dark areas were measured by the ImageJ software. Later diffusion processes may be involved in this darkening and may eventually lead to an overestimation of the electroporated area. Then we controlled that our initial clearer but visually evident electropermeabilized area were not significantly different from the darker one that was photographed some hours later. Experiments were repeated 3 times independently (N = 6).

Electric current density derived form electropermeabilization experiments was determined by the total flow of charge per time (A) over the cross section of area (*m* ^2^) at the end of each pulse. Cathodes were separated 0.5 cm from each other and immersed 1 cm in the tissue so, in an area very close to the central cathode (measurement area, indicated by an arrow in figures 1b) we assumed that we have a cross section of 1 cm×1 cm and that there it is present nearly all the total current. Electric conductivity was determined through the Ohm's law based on the applied voltage and the circulating electric current in this area at the end of each pulse.

### 0.2 *In silico* model

The three-dimensional mathematical model introduced here describes the electric field distribution by a nonlinear Laplace equation with variable conductivity depending on the electric field, the temperature and the number of pulses; and the Penne's Bioheat equation for temperature variations. The equations read:
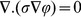
(1)

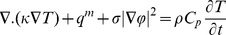
(2)

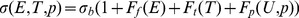
(3)being *φ* the electric potential, *κ* the thermal conductivity, *T* the temperature, *q^m^* the metabolic heat generation, *ρ* the tissue density, *C_p_* the tissue heat capacity and *t* the time. In living animal tissues there is a cooling effect derived from blood stream that is not present in potato tissue, so this contribution was eliminated from the bioheat equation in our case. The cooling effect is included in the system during the “off” time mainly by heat diffusion through tissue and needles. In the Comsol implementation it was also included convective and radiative cooling from the portion of the needles that is out of tissue to the environment.

Here, we introduced a new formulation for the electric conductivity *σ* in which we add a pulse-dependence. In this new formulation, the electric conductivity is dependent on three factors: the electric field (E), the temperature (T) and the number of pulses applied (p); where *σ_b_* is the basal electric conductivity of the tissue; and 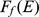, 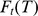 and 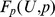, the terms dependent on the electric field, the temperature; and the applied voltage between electrodes (U) and number of pulses, respectively.


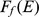
, based on [22], reads:
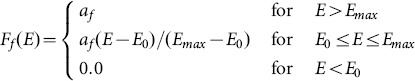
(4)being *a_f_* the electric field coefficient. 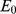 and 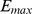 are defined in table 1.

**Table 1:** Input parameters of the numerical model.

Parameter	Value	Reference
*σ_b_*, basal electric conductivity	0.03 *S*/*m*	[22]
*κ*, thermal conductivity	0.562 *W*/*mK*	[41]
*q^m^*, metabolic heat generation	2161 *W*/*m* ^3^	[42]
*ρ*, tissue density	1100 *kg*/*m* ^3^	[43]
*C_p_*, heat capacity	3780 *J/kgK*	[41]
*T_p_*, physiological temperature	25 *C*	
*a_f_*, electric field coefficient	13.868	
*E*_0_	100 *V*/*cm*	
*E_max_*	1200 *V*/*cm*	
*a_t_*, temperature coefficient	0.135 1/*C*	[44]

*F_t_*(*T*), taken from [33], reads:
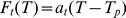
(5)being *a_t_* the temperature coefficient and 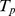 the physiological temperature.

Finally, 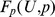, fitted to experimental measurements from potato tissue, reads:
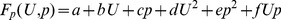
(6)with 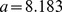, 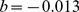, 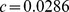, 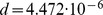, 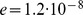 and 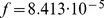. Electric conductivity derived from experimental data, after extracting the contribution of the terms that are function of the electric field and temperature, are approximated with the best 3-D fit to a second-order polynomial dependent on U and p using CurveExpert (version 2.0.4, www.curveexpert.net).

The system above was solved, for each time step, in a fixed domain on a three-dimensional variable triangular mesh using finite elements and deterministic relaxation techniques. The computational model was implemented in Fortran 90 and executed on a I7-class computer under Linux. The model was also solved using the commercial finite element package Comsol (Stockholm, Sweden) under Windows 7, simulating the application of the electric pulses through the addition of successive steps in which the applied voltage was switched on and off.

In the Comsol implementation, it is possible to add different packages according to the physics of the problem. Here, we used the Electric Currents and the Bioheat Transfer packages. The former solves the Laplace's equation (a steady-state equation but with parameters that are time-dependent, because they depend on the bioheat equation) and the latter, the Penne's bioheat equation (time-dependent). Both packages were solved time-dependently and were coupled through the electric field term of their respective equations. Up to eight electric pulses were simulated through 22 sequential time steps, each one with its own physical conditions. To avoid numerical errors due to different orders of magnitude in time lengths, it was necessary to use two steps for the “off” time between pulses. During the pulse application both packages are on, while during the time between pulses only the Bioheat Transfer package is present. Figure 1b shows the domain and mesh generated by Comsol. Table 1 shows the input parameters used in the numerical model. The model was calibrated with experimental measurements of electric currents in potato tissue during EP protocols.

## Results and Discussion

**Figure 2:**
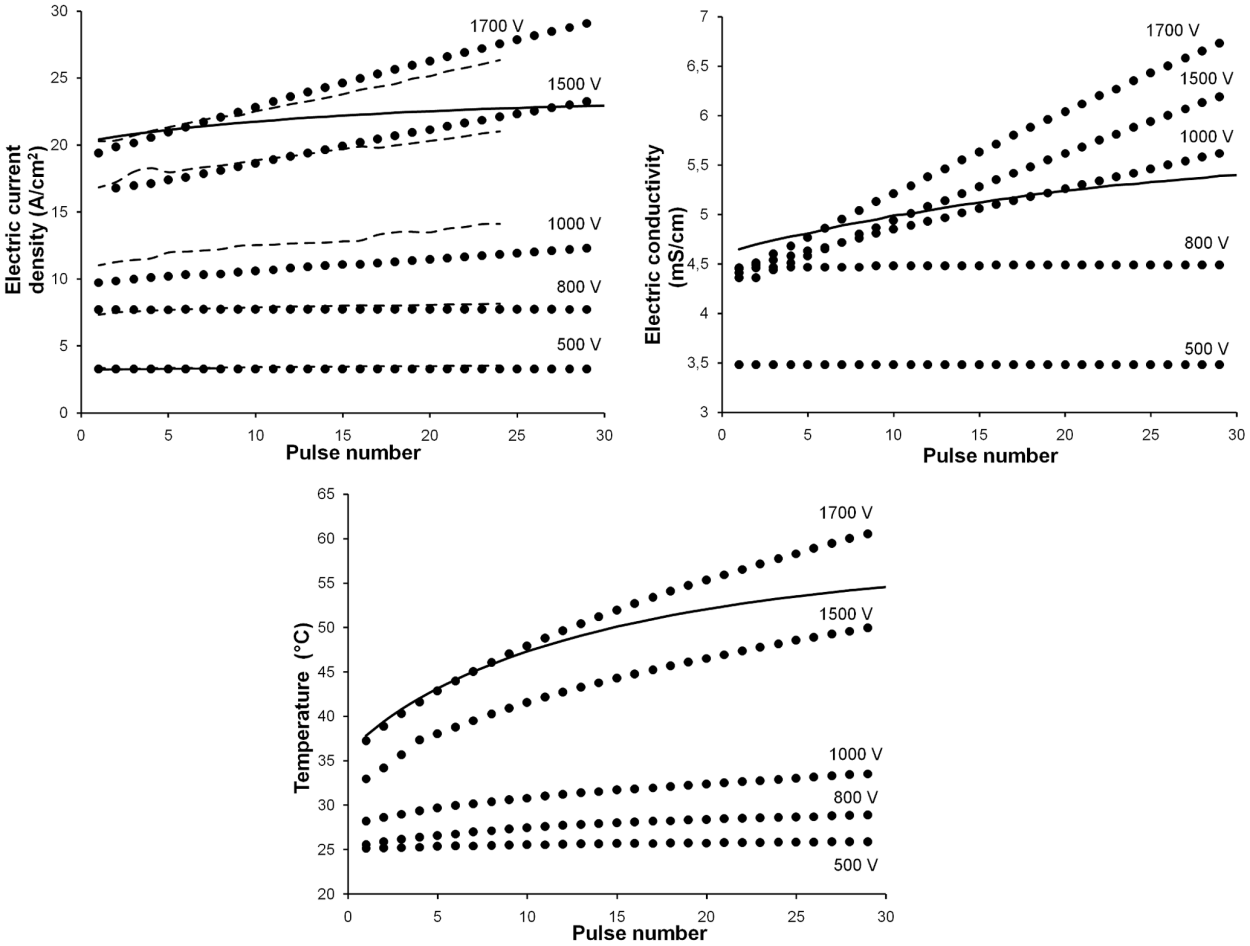
Temporal evolution of main variables. a) Electric current density (*A*/*cm* ^2^), b) electric conductivity (*mS*/*cm*) and c) temperature (*C*) vs. pulse number for different square electric pulses (voltages of 500, 800, 1000, 1500 and 1700 V; 100 *µ*s, 1 Hz). Circles: predicted values from the Fortran code. Dashed lines: experimental data from EP treatments in potato tissue. Standard errors were omitted for clarity. Solid lines: predictions from a model without the pulse-conductivity term for 1700 V.

**Figure 3:**
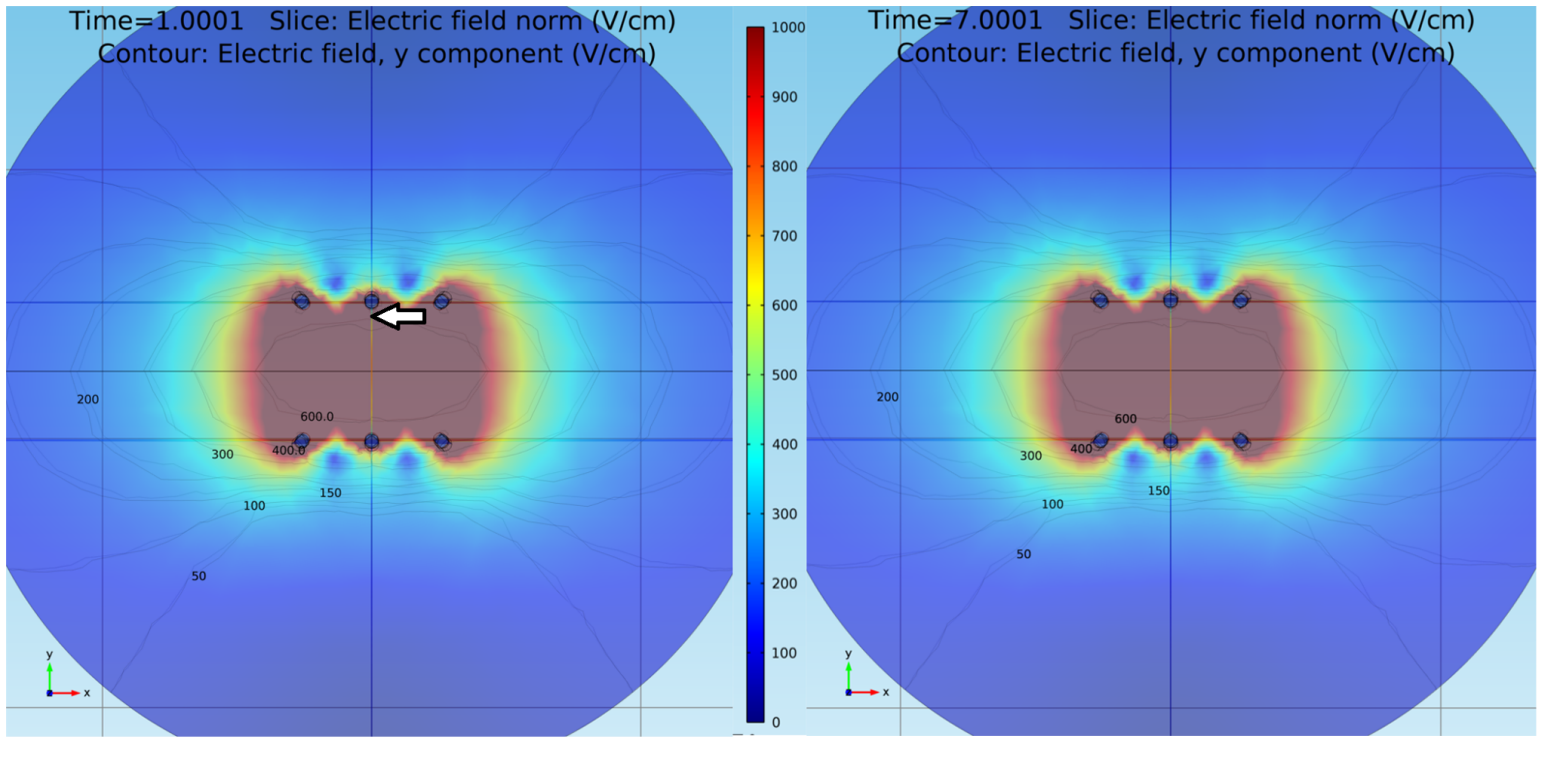
Electric field distribution. Predicted electric field distribution (*V*/*cm*) in the x-y plane generated by Comsol after an EP treatment with a) 2 and b) 8 square electric pulses of 1500 V, 100 *µ*s and 1 Hz. Isolines correspond to 50, 100, 150, 200, 300, 400 and 600 V/cm. The arrow indicates the point where all calculations presented in figure 2 were made (measurement area).

Figure 2 shows experimental measurements on potato tissue and theoretical predictions (with or without the pulse-dependent conductivity term) of tissue electric current density, electric conductivity and temperature as a function of the number of pulses, for different applied voltages. In all cases, 100 *µ*s pulses were delivered at 1 Hz through the described electrode configuration. All data presented in this figure (experimental as well as numerical) correspond to the end of each pulse. Experimental data (figure 2a, dashed lines) show that, at applied voltages higher than 1000 V, electric current density (thus electric conductivity) increases with a higher number of pulses (linear regression is significant for 1000, 1500 and 1700 V). For a better comparison between experimental and numerical results, we chose to analyze electric current density in place of total electric currents. Electric current density is not homogeneous in the electropermeabilized tissue [36], as neither is the electric field, the conductivity nor the temperature; so we calculated and reported all these variables in a defined area close to the central cathode (measurement area, indicated by an arrow in figures 1b and 3a).

Theoretical predictions with the pulse-dependent conductivity term (circles) describe how, at applied voltages higher than 1000 V, electric current density (figure 2a), conductivity (figure 2b) and temperature (figure 2c) rise during the train of pulses. It is worth noting that, under certain EP conditions, the influence of pulses on temperature is considerable. This is explained theoretically by the fact that conductivity strongly affects the Joule heating term in the Penne's Bioheat equation. During the inter-pulse (“off”) time, there is a cooling effect determined by heat diffusion and, in the case of the Comsol implementation, also convective and radiative cooling to the environment. Nevertheless this effect is not enough to cool completely the system before the arrival of the next pulse. Other groups have already reported the influence of pulse number on temperature rises in an ECT [37] and an irreversible electroporation [21] context.

Numerical predictions with the pulse-dependent conductivity term are quantitatively close to experimental results of electric current density, both regarding initial values and slopes (figure 2a). In contrast, predictions obtained without this term (solid lines corresponding to 1700 V) compare unfavourably with experimental measurements. They do not reflect either the correspondent increment in electric conductivity (figure 2b) and present lower temperature estimations (figure 2c).

All results presented in figure 2 were derived from the Fortran code. In this figure, Comsol curves were omitted for clarity, but results obtained with the Comsol software were very similar (concerning both origin ordinates and slopes) to those obtained with Fortran for the electric current density, electric fields and conductivity; being slightly lower in the case of temperature predictions for 1500 and 1700 V (data not shown). Perhaps this last divergence may be attributed to the inclusion of convective and radiative cooling in the case of the Comsol implementation.

In previous Comsol EP modeling present in literature, there is not a real inclusion of individual electric pulses [33],[34]. In these papers, instead of modeling 80 pulses and in order to avoid numerical problems, they modified the approach and, to deliver the same amount of energy as in the pulsed approach, multiplied the Joule heating by the duty cycle (duration/period) of the pulse in the tissue and insulation domains. Although this approximation is correct, especially considering applications of high quantity of pulses, our approximation is closer to real conditions as we actually model the pulses. With the implementation of our series of sequential time-dependent 22 steps described above to simulate 8 pulses, we avoid abrupt changes and numerical errors through the application of a variable time discretization and the use of two different time-scales during the “off” time. In this manner we managed to acquire an adequate definition and capture of the microsecond pulses by the solver.

Figure 3 presents Comsol theoretical results describing the distribution of the electric field at the end of the second pulse (figure 3a) and the eighth pulse (figure 3b) of an EP protocol with pulses of 1500 V, 100 *µ*s and 1 Hz. Contrary to what was expected, although there are small local changes in the electric field intensity values, these changes do not induce significant changes in the averaged electric field distribution. These results were corroborated with those obtained with the Fortran code and are valid in the context of relatively homogeneous tissues as potato tissue. Future experimental and numerical studies would be necessary to fully adjust these analysis to more inhomogeneous and complex tissues as animal tumors.

**Figure 4:**
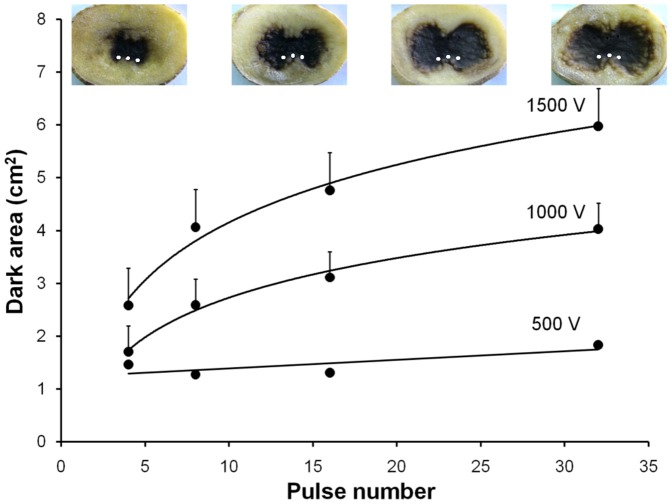
Electropermeabilized area. Dark (electropermeabilized) potato area (*cm* ^2^) vs. pulse number for different EP protocols (4, 8, 16 and 32 square pulses of 100 *µ*s, 1 Hz with amplitudes of 500, 1000 and 1500 V). Bars: standard errors. Lines: logarithmic regressions. Images at the top of the figure correspond to potato tissue treated with 4, 8, 16 and 32 pulses of 100 *µ*s, 1 Hz and 1500 V. Cathode position is indicated as white spots.

Figure 4 shows experimental measurements and images of the blackened (electropermeabilized) potato area vs. pulse application for different applied electric voltages. The spatial position of the three cathodes is indicated as white spots in the photographs for an easier comparison with figure 3. It is observed that, at constant voltages higher than 1000 V, the dark area increases with the number of pulses (data best fit is to a logarithmic regression). To explain this we propose that, although the electric field distribution is strongly related to the permeabilized area, it is not the unique factor responsible of it. Electric currents and tissue conductivity could be important as well.

Mir reported that tissue conductivity changes could be important in the treatment outcome [15], that the amount of permeabilization would depend on the conductivity of the extracellular medium, and that if this conductivity decreases then it would be necessary to deliver a pulse of larger amplitude in order to achieve the same effect [16]. Miklavcic also observed, at pulses of 1500 V, significant changes of liver tissue electrical conductivity as well as electric current rises during the sequence of pulses with no significant changes in the electric field distribution [38]. On the other hand it was experimentally established that, for certain EP protocols, pulse addition may rise electric conductivity [15] and may even affect the amount of permeabilization [17],[18] probably due to, at least in part, the formation of more and/or larger stable transport pores in the plasmatic membrane [20]. Rols et al. [17] showed that cells may be permeabilized at lower electric field intensities if the medium ionic strength increases and that the derivative of the extent of permeabilization versus the electric field intensity, the pulse duration or the number of pulses is also function of the ionic strength.

Based on these reports and on our own results, we hypothesize that the addition of pulses during the treatment progressively increases tissue conductivity (along with electric currents) making cells more susceptible to permeabilization by the electric field. This fact may explain the enlargement of potato electroporated area observed in figure 4 mainly by an increment in tissue conductivity and circulating electric currents, without a strong change in the electric field distribution. In addition to a rise in membrane conductance and cell recruitment, other electrochemical tissue changes related to the extracellular matrix compartment could also be involved in these pulse-dependent conductivity rises. The application of high voltage electric pulses may be increasing tissue ionic strength by a rise in the ionic concentration of the extracellular matrix (ECM) and/or a rise in the ionization state of ECM or membrane-anchored macromolecules (mainly proteins, proteoglicans and glycoproteins). These ionic changes induced by high electric fields in the ECM would not be completely reversed during the “off” time between pulses, so tissue conductivity may slowly increase from pulse to pulse. Nevertheless this point is subject of further studies.

In this work a frequency of 1 Hz was considered due to its standard use in ECT clinical applications, but other studies have addressed the effect of pulse frequency on ECT effectiveness. Application of higher pulse frequencies (higher than those causing tetanic contraction) would have the advantage of reducing muscle contractions and related unpleasant sensations during the treatment. In vitro studies have determined that cell uptake in response to ECT does not change between 1 Hz to 8.3 kHz [39] and these results were corroborated with in vivo studies using optimal drug doses [40]. This fact would favor the use of higher pulse frequencies in ECT clinics. Nevertheless, when suboptimal doses are applied, greater uptake effectiveness was observed at 1 Hz than at 5 kHz [40]. At higher pulse frequencies, as for 1 kHz, the effect on tissue temperature rise is also considerable [37].

In summary, experimental measurements in potato tissue show that, during a train of pulses and keeping constant the applied voltage, electric current density and the blackened (electropermeabilized) area may increase. Our theoretical predictions agree with these experimental results. In particular, they confirm previous data that electric currents may increase during an ECT treatment at high voltage electric pulses with no significant changes in the electric field distribution. This would imply a rise in tissue electric conductivity, in turn induced by the pulse number and the temperature.

## Conclusions

We presented experimental measurements from a vegetable tissue model showing electric currents and tissue electropermeabilized area for different EP protocols revealing that, with increased number of pulses and keeping constant the applied electric voltage, the current density and the blackened (electropermeabilized) area increase. We also presented a new theoretical model describing the electric field distribution with a variable conductivity coefficient depending on the electric field, the temperature and the number of pulses applied. Theoretical predictions come close to experimental measurements and explain the current rise observed at high-voltage electric pulses by an increment in the electric conductivity with no significant changes in the electric field distribution. This in turn may be induced by the addition of pulses and the temperature rise. A direct consequence of these results is that, under certain conditions and taking into account the temperature, it is possible to obtain larger electropermeabilized areas with the same voltage applied by increasing the pulse number. The development of more realistic mathematical models as the one presented here will hopefully contribute to optimize experimental EP protocols and to predict better their treatment outcomes.
